# Mesenchymal Stem Cells and the Origin of Ewing's Sarcoma

**DOI:** 10.1155/2011/276463

**Published:** 2010-10-05

**Authors:** Patrick P. Lin, Yongxing Wang, Guillermina Lozano

**Affiliations:** ^1^Department of Orthopaedic Oncology (Unit 1448), University of Texas MD Anderson Cancer Center, 1515 Holcombe Boulevard, Houston, TX 77030, USA; ^2^Department of Genetics (Unit 1010), University of Texas MD Anderson Cancer Center, 1515 Holcombe Boulevard, Houston, TX 77030, USA

## Abstract

The origin of Ewing's sarcoma is a subject of much debate. Once thought to be derived from primitive neuroectodermal cells, many now believe it to arise from a mesenchymal stem cell (MSC). Expression of the *EWS-FLI1* fusion gene in MSCs changes cell morphology to resemble Ewing's sarcoma and induces expression of neuroectodermal markers. In murine cells, transformation to sarcomas can occur. In knockdown experiments, Ewing's sarcoma cells develop characteristics of MSCs and the ability to differentiate into mesodermal lineages. However, it cannot be concluded that MSCs are the cell of origin. The concept of an MSC still needs to be rigorously defined, and there may be different subpopulations of mesenchymal pluripotential cells. Furthermore, *EWS-FLI1* by itself does not transform human cells, and cooperating mutations appear to be necessary. Therefore, while it is possible that Ewing's sarcoma may originate from a primitive mesenchymal cell, the idea needs to be refined further.

## 1. Introduction

Ewing's sarcoma is a rare malignancy primarily affecting children and adolescents. It arises mainly in bone and less commonly in soft tissues. The poorly differentiated tumors are aggressive and metastasize early to lung, bone marrow, and other tissues [1–4]. In all cases of the disease, there is a characteristic reciprocal chromosomal translocation, which leads to an in-frame fusion between the *EWS* gene and one of the *ETS* family gene members [5, 6]. In approximately 85% of cases, the *EWS* gene is combined with the *ETS* gene *FLI1* in a t(11;22) translocation. In about 10% of cases, *EWS* is fused to *ERG*, which has a high degree of homology with *FLI1* in the carboxyl terminus. In very rare cases, *EWS* is fused to other *ETS* family genes such as *FEV *and* ETV1* [1, 7]. The *EWS-ETS* fusion gene appears to be critically important for maintaining the tumor phenotype of the disease. Ewing's sarcoma cells with *EWS-FLI1* knockdown by siRNA exhibit decreased cell proliferation, and tumor xenografts regress in mice [8, 9]. 

## 2. Mechanism for *EWS-FLI1* in Tumorigenesis

The mechanism by which EWS-FLI1 contributes to tumorigenesis is complex since the gene affects the cell in many different ways. The best known function of the EWS-FLI1 protein is that of an aberrant transcription factor [10]. Its structure consists of an N-terminal *EWS* transcriptional activation domain and a C-terminal *FLI1* DNA-binding domain. Disruption of either domain impairs the transformation activity of the protein [11, 12]. 

*EWS-FLI1* possesses the winged helix-turn-helix motif of the *ETS *family of transcription factors, and EWS-FLI1 recognizes the same consensus DNA binding sequence as *FLI1*. However, because of aberrant protein-protein interactions, in large part due to the EWS portion of the molecule, *EWS-FLI1* has an altered profile of gene regulation. It is relevant to note that *FLI1* can act as an oncogene in its own right. *Fli1* (Friend leukemia virus integration) was originally identified as the gene activated by insertion of the Friend murine leukemia virus (MuLV) [13]. It has the capacity to transform murine cells and is responsible for the development of erythroleukemias in mice [14]. It is presumably the fusion of the N-terminal moiety of *EWS* that confers the ability to induce Ewing's sarcoma in human cells. 

Acting as either a transcriptional activator or repressor, EWS-FLI1 regulates a number of important target genes. EWS-FLI1 has been found to upregulate genes that promote cell survival and proliferation, including *IGF1* [15],* Myc* [16], *TOPK* [17], *NKX2.2* [18], *ID2* [19], *DAX1* [20], *GLI1* [21], *EZH2* [22], *MK-STYX *[23], and *PLD2* [24]. EWS-FLI1 concomitantly represses genes that induce cell cycle arrest and apoptosis, including *TGFB2 *[25], *p21* [26], *p57kip* [27], and *IGFBP3* [28]. Interestingly, EWS-FLI1 also upregulates genes that are involved in cell differentiation such as *SOX2 *[29] and *EZH2* [22]. 

While progress has been made in characterizing the downstream targets of EWS-FLI1, the properties of EWS-FLI1 may not be fully explained on the basis of its activity as a transcription factor. There are other properties of EWS-FLI1 that have not been studied quite so extensively. These include RNA binding, RNA splicing and protein-protein interactions [30–37]. Furthermore, it has become increasingly clear that the cellular context has a large bearing upon the transformation process. Early experiments with *EWS-FLI1* in NIH3T3 cells were somewhat artificial in the sense that these cells were immortalized murine fibroblasts. Paradoxically, in normal murine and human fibroblasts, EWS-FLI1 by itself does not transform cells; instead, it results in cell cycle arrest [38, 39]. These findings suggest the possibility that EWS-FLI1 may have complex antagonistic effects on different types of cells and underscore the importance of identifying the correct cell of origin for the disease.

## 3. Primitive Neuroectodermal Features of Ewing's Sarcoma

The origin of Ewing's sarcoma is a controversial topic [7]. Histologically, Ewing's sarcoma has a certain resemblance to primitive neuroectodermal cells, and it was once widely believed that the tumor arose from such cells [40, 41]. Early neural markers, such as neuron-specific enolase (NSE) and S-100, are present in some tumors [42, 43]. Ultrastructural features, such as neurosecretory granules, can also be observed with electron microscopy in some cases as well [41, 43]. Laboratory experiments have demonstrated that Ewing's sarcoma cells can be induced to differentiate towards the neural lineage *in vitro *and acquire neuritic processes under appropriate stimulation [44]. Conversely, introduction of EWS-FLI1 into neuroblastoma cell lines has been shown to make the cells less differentiated and acquire characteristics of Ewing's sarcoma [45]. 

In spite of considerable data pointing to a neuroectodermal origin, some doubt has been cast upon this idea. The neural markers seen in Ewing's sarcoma are present only in a minority of cases, and even in such tumors, they occur sporadically in cells. More importantly, *EWS-FLI1* has been shown to induce neuroectodermal differentiation and upregulate a number of genes associated with early neural differentiation [46, 47]. The findings raise the possibility that the neuroectodermal characteristics of Ewing's sarcoma might be a direct result of *EWS-FLI1* expression and not necessarily the cell of origin.

## 4. Mesenchymal Stem Cells

An alternative hypothesis is that Ewing's sarcoma derives from a mesenchymal stem cell. This addresses one simple but nagging question regarding the neuroectodermal theory of histogenesis, namely, whether bone normally contains primitive neuroectodermal cells. While cranial bones develop from mesenchymal condensation of neuroectoderm [48], the long bones of the limbs originate from mesoderm [49], and there may not normally be primitive neuroectodermal cells in bone. Since most cases of Ewing's sarcoma arise in bone, it seems plausible that the cell of origin ought to be a normal resident of bone. It may be significant to note that mesenchymal cells in bone marrow can exhibit some characteristics of neuroectodermal cells [48, 50]. The cells sometimes express neural markers spontaneously, including S-100 and neurofilament M [50]. They can also be induced *in vitro* to differentiate towards the neural lineage [51, 52]. 

A fundamental problem with the mesenchymal hypothesis is that one must define what exactly constitutes a “mesenchymal stem cell.” The term is widely used in scientific literature, and yet the existence of the cell has not been rigorously proven or defined [53]. The term MSC usually implies a bone marrow-derived cell that has the capacity to differentiate towards various mesodermal lineages, such as osteoblasts, chondrocytes, and adipocytes. In order to satisfy the criteria for being a stem cell, it must be shown to be (1) self-renewing and (2) able to generate different tissue types *in vivo* from a single cell. This has proven to be a daunting task, and perhaps as a result, the putative MSC has yet to be defined in terms of a reliable set of cell surface markers that would aid greatly in consistent extraction of the cells [54]. Cells considered to be MSCs have been reported to be positive for a number of markers, including Sca-1, Stro-1, SH2, SH3, SH4, CD29, CD44, CD90, and CD105, but none of these are entirely specific for MSCs [53–58]. 

Most preparations of MSCs are heterogeneous [59]. Bone marrow extracts are typically plated on plastic dishes, and the adherent cells are enriched for cells that have some of the desired properties of MSCs. However, the cultures contain a mixture of cells, including hematopoietic stem cells, endothelial cells, osteoblasts, and other cells. The cells can be stimulated to undergo differentiation towards osteoblastic, chondroblastic, and adipocytic lineages *in vitro* under appropriate culture conditions. One notes though that unless cultures are derived from single cell colonies, it is possible that different cell subpopulations differentiate into the various lineages, and it is therefore not accurate to call the cells MSCs. Furthermore, expression of *in vitro* markers of differentiation does not equate to the capacity for *in vivo *differentiation [53]. For these reasons, some authors prefer to call these cell preparations by less presumptive terms, such as “bone marrow stromal cells” as opposed to MSCs. 

There are data to support the idea that there is more than one type of pluripotential nonhematopoietic cell in bone marrow. Colter et al. showed that within the population of plastic adherent cells, there are at least two distinct subgroups which could be separated on the basis of size and cell surface markers [60]. A small, round cell with rapid doubling time seems to predominate at early passage whereas a larger spindle cell tends to divide more rapidly at later times. The latter cell type may be an osteoblast precursor and perhaps less pluripotential since it spontaneously expresses alkaline phosphatase, an osteoblast marker. 

Jiang et al. showed that bone marrow contains a small population of pluripotential cells, which they termed “marrow-associated progenitor cells” (MAPCs) [61]. The cells represent a subset of the bone marrow cells that adhere to plastic. The cells were found to have clonogenic self-renewal capabilities. Furthermore, when injected into blastocysts, the cells developed into many different tissues, including those derived from ectoderm, endoderm, and mesoderm. These cells may be distinct from the majority of cells typically termed MSCs since their cell surface markers appear to be different from most of the plastic-adherent cells extracted from bone marrow. 

At the present time, it should be recognized that our understanding of the “mesenchymal stem cell” is far from complete. The exact nature and composition of the majority of plastic-adherent bone marrow cells is still in question. The very primitive stem cell described by Jiang et al. [61] may be similar to the stem cells found in other tissues [62] and may not represent the more prevalent bone marrow stromal cell. Within the latter group of cells, there may be multiple sub-populations of cell types with varying pluripotential capabilities for *in vivo *differentiation, analogous to hematopoietic cells [54].

## 5. MSC Characteristics of Ewing's Sarcoma

There are several lines of evidence that support the notion that Ewing's sarcoma is derived from an MSC-like cell (accepting for the moment the concept of the MSC). Using Ewing's sarcoma cell lines, researchers have knocked down the expression of *EWS-FLI1* and shown that the cells have some capacity for *in vitro* differentiation towards chondroblastic, osteoblastic, and adipocytic lineages [63]. Several groups have demonstrated that expression of *EWS-FLI1* in murine MSCs resulted in transformation of the cells, and when these cells were implanted into mice, sarcomas formed [64, 65]. The tumors shared some characteristics with Ewing's sarcoma, including cell surface markers and cell morphology. In a related set of experiments, expression of *EWS-FLI1* in the pluripotential murine cell line C3H10T1/2 inhibited the cells ability to differentiate into osteoblasts and adipocytes while upregulating neural genes [66]. When injected into mice, these cells formed metastatic sarcomas. 

In contrast to the mouse studies, human MSCs that were infected with a retrovirus containing *EWS-FLI1* failed to form tumors when injected into immunodeficient mice [67]. Although somewhat disappointing, the results are not at all surprising since it has been reported that transformation of normal human mesenchymal cells requires multiple mutations [68]. Murine mesenchymal cells are more apt to transform spontaneously in culture and form sarcomas [69]. It is worthwhile to note that human cells expressing *EWS-FLI1* take on a rounded morphology and express some of the neuroectodermal markers seen in Ewing's sarcoma [67, 70]. Furthermore, the expression of CD99, a relatively specific marker for Ewing's sarcoma, was found to be present at a low level in MSCs and upregulated by *EWS-FLI1* [67]. This addressed a previously raised concern against the theory of an MSC origin of Ewing's sarcoma regarding expression of CD99 in MSCs [71]. 

The relationship between Ewing's sarcoma and human mesodermal cells has been strengthened further in other experiments. Ewing's sarcoma cell lines with knockdown of *EWS-FLI1* have a transcription profile similar to that of the human fetal fibroblast cell line IMR-90 [75]. Similarly, Tirode et al. showed that in an *EWS-FLI1* knock-down experiment, the gene expression profile of the cells began to converge upon that of mesenchymal stem cells [63]. This was statistically significant for the subset of genes that were upregulated or downregulated by *EWS-FLI1*, but it is noteworthy that the overall pattern of gene expression appeared to have distinct differences between MSCs and Ewing's sarcoma cells with *EWS-FLI1* knockdown. Comparison of cell surface markers has also shown some similarities and differences between MSCs and Ewing's sarcoma (Table 1). A complementary set of observations was made by Burns et al., who reported that late passage human mesenchymal cells that spontaneously transformed after introduction of telomerase (hMSC-TERT20) took on an immunohistochemical profile that was reminiscent of Ewing's sarcoma, namely, CD99+, vimentin+, CD45−, cytokeratin−, and desmin− [72]. Finally, in a meta-analysis of studies on genes affected by *EWS-FLI1*, a “core *EWS-FLI1* transcriptional signature” was identified which shared similarities with published mesenchymal stem cell data [76]. While these collective data are supportive of a mesenchymal origin of Ewing's sarcoma, it is clear that simple knock-down of *EWS-FLI1* in tumor cells does not cause them to revert to a normal mesenchymal cell. Other changes have accumulated in the cells that make them distinctly different from recognizable normal cells.

## 6. Knock-in and Transgenic Mouse Models

The importance of cellular context has been highlighted in recent mouse models. Several knock-in and transgenic mice expressing EWS-FLI1 have now been created. In all of these models, a strategy of conditional expression has been employed to avoid lethal effects of *EWS-FLI1*. Constitutive expression of EWS-FLI1 protein in embryonic stem cells causes cell death [77], and mice with expression of EWS-FLI1 in the whole body have an embryonic lethal phenotype [78, 79]. To circumvent this problem, investigators have used c*re-loxP* recombination to achieve conditional expression of the protein in vivo. Tissue-specific *cre *transgenic mice are crossed to the *EWS-FLI1* mice to restrict expression of the protein to certain tissues or cells.

Torchia et al. developed a knock-in *EWS-FLI1 *mouse at the Rosa26 locus [78]. When *EWS-FLI1* was expressed by use of the *Mx1-cre* mouse, myeloid/erythroid leukemias developed. These malignancies were similar to but distinct from the erythroleukemias induced by F-MuLV. The *Mx1-cre* mouse expresses *cre* recombinase in the liver, spleen, bone marrow, and lymphoid tissues after induction with alpha/beta interferon or polyinosinic*poly(C) (pIpC) [80]. While the original strategy was to target expression to bone marrow progenitor cells, it is likely that the gene was preferentially expressed in hematopoietic precursors, thereby producing leukemias. 

Codrington et al. developed an interesting *Ews-ERG* mouse, which possesses an inverted ERG cassette flanked by loxP sites in intron 8 of the *Ews* gene, to simulate the t(21;22) translocation that produces the *EWS-ERG* fusion [81]. They obtained T cell lymphomas in all mice but this may have been due to their choice of the *Rag1-cre* mouse, which expresses *cre* recombinase in lymphocytes only [82]. 

Although it may be somewhat surprising that leukemias and lymphomas were found in the previous two mouse models, there is reason to believe that this has clinical relevance to human disease. While it was once believed that *EWS-FLI1* was specific to Ewing's sarcoma, it is now known that *EWS-FLI1* and related *EWS-ETS* fusions occur sporadically in other malignancies, including leukemias [83–87] and biphenotypic tumors, which have features of both myogenic and neuroectodermal differentiation [88]. These cases indicate that *EWS-FLI1* can potentially contribute to neoplastic growth outside of the typical cellular context in which Ewing's sarcoma arises. Quite interestingly, the CD99 marker, which is commonly used for immunohistochemical testing for Ewing's sarcoma, is actually considered a normal leukocyte marker and is uniformly expressed on thymocytes [89].

In a different transgenic mouse model of *EWS-FLI1*, conditional expression of the fusion protein was achieved in the mesoderm-derived tissues of the limbs by crossing to the *Prx1-cre* mouse, which expresses *cre* in the primitive mesenchyme of the early limb bud [79]. Part of the rationale behind this cross was to spare the hematopoietic cells and thereby increase the likelihood of inducing sarcomas. Limb shortening, muscle atrophy, osseous dysplasia, and other developmental abnormalities were observed, reinforcing the idea that *EWS-FLI1* impairs growth and differentiation of cells. However, in this model, sarcomas did not spontaneously form unless the *p53* gene was simultaneously mutated [79]. In contrast to the more differentiated osteosarcomas that formed without *EWS-FLI1* in mice with conditional mutation of *p53* induced by *Prx1-cre*, the tumors that arose with *EWS-FLI1* were undifferentiated sarcomas, similar to Ewing's sarcoma. 

**Figure 1:**
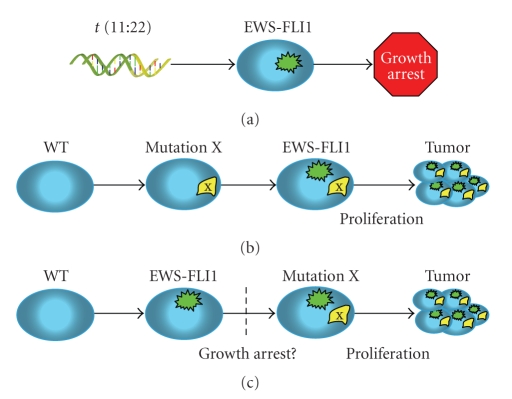
Cooperative mutations in the development of Ewing's sarcoma. (a) A t(11;22) reciprocal translocation produces the *EWS-FLI1* gene, but this tends to cause growth arrest in normal cells. (b) A mutation randomly occurring prior to the t(11;22) translocation might cooperate with *EWS-FLI1 *to permit escape from growth arrest (or even promote cell proliferation) and subsequent transformation to Ewing's sarcoma. (c) The cooperative mutation may occur after the t(11;22) translocation; this would necessarily imply a mechanism for continued cell growth after *EWS-FLI1* is expressed.

Although concern has been raised regarding the validity of mouse models for Ewing's sarcoma [76], the transgenic mouse model employing *Prx1-cre* does underscore one important feature of Ewing's sarcoma, namely, that *EWS-FLI1* alone does not appear to be sufficient to confer sarcomatous change in an *in vivo* setting. Similar to the results obtained with human MSCs expressing *EWS-FLI1, *additional cooperating mutations seem to be required for transformation to occur. The nature and timing of these mutations may be an interesting subject for future research (Figure 1). The limb shortening of transgenic mice [79] and experimental cell culture work of various groups [39, 50] suggest that *EWS-FLI1 *impairs growth of normal cells, and this may be problematic in envisioning a pathway for neoplastic transformation to occur. While many have postulated that mutations occur after the t(11;22) translocation event, it is also possible that *EWS-FLI1* transforms a cell that carries a silent but critical mutation in another gene. 

The requirement for additional mutational events has precedent in other mouse models. For example, in a knock-in model of alveolar rhabdomyosarcoma, mutations in *Ink4A/Arf* or *Trp53* were needed for the *Pax3;Fkhr* fusion to transform cells effectively [90]. Interestingly, conditional expression in the correct cell of origin—postnatal differentiating myofibers—was also found to be important for rhabdomyosarcomas to develop in this particular model. *TRP53,* however, is unlikely to be the critical cooperating mutated gene in Ewing's sarcoma since TRP53 mutations are found only in about 10% of Ewing's sarcoma cases [91].

## 7. Conclusions

Identification of the correct cell of origin is crucial to the understanding of Ewing's sarcoma. While there has been steadily accumulating evidence that the cell of origin may be a primitive pluripotential cell that resides in bone marrow, one cannot state unequivocally that it is either a neuroectodermal cell or a “mesenchymal stem cell.” One important simple reason for this is that the concept of the MSC has yet to be rigorously defined, and the term is used somewhat loosely in the scientific literature. MSCs, as they are typically prepared, are composed of a mixture of different cells, and some of these do exhibit neuroectodermal properties. It is possible that a subpopulation of the pluripotential cells in bone marrow is especially vulnerable to the transforming effects of *EWS-FLI1*. 

Even if the correct cell of origin can be identified with precision, much has yet to be learned. Expression of *EWS-FLI1* alone is not sufficient for transformation of normal human cells. Instead, *EWS-FLI1* tends to cause growth arrest. In the early excitement over the discovery of *EWS-FLI1*, researchers focused largely upon downstream transcriptional targets. Current research suggests that there may be critically important cooperating mutations that work in concert with *EWS-FLI1*. The “cell of origin” concept may be a simplistic one in that it might not be a normal cell from which Ewing's sarcoma arises. *EWS-FLI1* may be fully transforming only in a mutated cell that possesses additional genetic alterations. The tumor that eventually arises, whether it is a conventional Ewing's sarcoma, an unusual variant, a biphenotypic tumor, lymphoblastic leukemia, or some other disease, will probably depend upon an interplay between the cellular background in which it arises and the set of cooperating mutations that occur.

## Figures and Tables

**Table 1:** Cell surface marker analysis of mesenchymal cells and Ewing's sarcoma.

Marker	Mes. Cells	Mes. Cells + EWS-FLI1	ESFT	ESFT + EWS-FLI1 knockdown	Refs.
CD10	+	−	−		[70]
CD13	+	−	−		[70]
CD29	+		−	+	[63]
CD44	+	+	−	+	[57, 63, 70]
CD45 (leukocyte common antigen)	−		−		[57, 72]
CD54	∓	+	∓	+	[63, 70]
CD59	+		−	+	[63]
CD73	+		−	+	[63]
CD99 (MIC2)	∓	+	++	+	[67, 70, 71, 73]
CD105	+	+	∓	+	[57, 63, 70]
CD 117 (c-kit)	−	+	+		[70]
CD166	+	+	+	+	[63, 70]
CD271	−	+	+		[70]
Vimentin	+		+		[72]
Caveolin-1	+		+		[73, 74]
Desmin	−		−		[72]
Cytokeratin	−		−		[72]

Mes. cells: human bone marrow-derived mesenchymal cells.

ESFT: Ewing's sarcoma family of tumors.

∓ indicates weak or variable staining.
